# Satellite DNA Editing Enables Multiplexed Chromosome Restructuring in *Populus*

**DOI:** 10.1111/pbi.70741

**Published:** 2026-08-24

**Authors:** Ran Zhou, Margot S. S. Chen, MaKenzie R. Drowns, Kathrine Mailloux, Kangquan Yin, Brieanne Vaillancourt, C. Robin Buell, Chung‐Jui Tsai

**Affiliations:** ^1^ Warnell School of Forestry and Natural Resources University of Georgia Athens Georgia USA; ^2^ Department of Genetics University of Georgia Athens Georgia USA; ^3^ Department of Plant Biology University of Georgia Athens Georgia USA; ^4^ The Plant Center University of Georgia Athens Georgia USA; ^5^ Center for Applied Genetic Technologies University of Georgia Athens Georgia USA; ^6^ Institute of Plant Breeding, Genetics and Genomics Athens Georgia USA; ^7^ Department of Crop and Soil Sciences University of Georgia Athens Georgia USA

Chromosome engineering can reshape genome architecture to alter recombination, gene dosage, or karyotype, with clear relevance to crop improvement (Puchta and Houben 2024). In plants, however, chromosome‐scale engineering typically relies on targeted double‐strand breaks (DSBs) and sexual reproduction to recover intra‐ or interchromosomal rearrangements (Beying et al. 2020; Chen et al. 2026; Rönspies et al. 2025, 2022; Schmidt et al. 2020; Schwartz et al. 2020; Zhao et al. 2025). In perennial species with long generation times, this strategy remains impractical. Here, we show that CRISPR targeting of abundant satellite DNA enables multiplexed chromosome restructuring in aspen ( *Populus tremula* × *alba*; INRA 717‐1B4, hereafter “717”), generating diverse large‐scale structural variants (SVs) in primary transformants.

We leveraged CRISPR–Cas9 for high‐plex editing of M147, an abundant, non‐centromeric satellite DNA organised in tandem repeat arrays (TRAs) on most chromosomes in 717 (Zhou et al. 2023). A single guide‐RNA (gRNA) targeting the consensus sequence can induce DSBs at numerous loci genome‐wide. Combined with tissue culture regeneration, this approach enables chromosome‐scale engineering without sexual reproduction (Figure 1A). We tested this approach using two gRNA designs, M147.g7 and M147.g8, with 47,965 and 29,853 predicted targets, respectively (Table S1). Stable transformation by *Agrobacterium tumefaciens* yielded 14 and 27 independent callus lines for M147.g7 (trial #7) and M147.g8 (trial #8), respectively. Of these, only two and three regenerated into whole plants, well below typical recovery rates (Bewg et al. 2022). Callus recovery was lower for M147.g7 with more predicted target sites, suggesting reduced tolerance to extensive genome cleavage. A second transformation with M147.g8 (trial #9) using triple the number of leaf‐disc explants yielded 50 calli and 11 independent plant lines (Table S2), demonstrating reproducible, albeit low, transformation efficiency.

**FIGURE 1:**
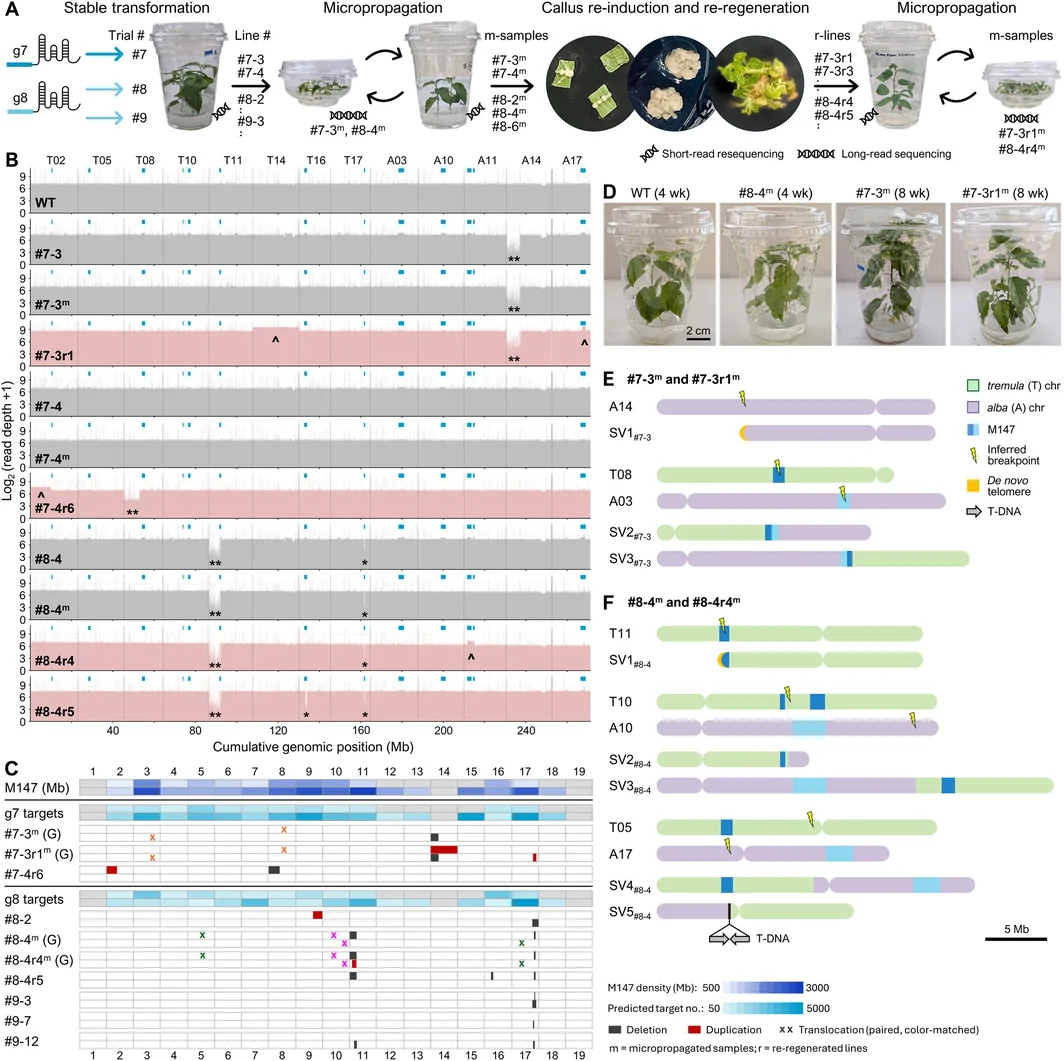
Genome‐wide structural variation in transgenic *Populus*. (A) Overview of transgenic lines and sampling scheme. (B) Resequencing read coverage (log_2_) for affected chromosomes in WT and transgenic lines (m, micropropagated; r, re‐regenerated). Blue bars denote M147‐TRAs (*, deletion; **, arm‐loss; ^, duplication). (C) Summary of structural variants (SVs) across *tremula* (top) and *alba* (bottom) chromosomes from short‐read mapping or long‐read genome assemblies [G]. Heatmaps show M147 abundance (dark blue) and target density (light blue); deletion (black), duplication (red), translocation (paired ×). (D) Representative tissue culture plants. (E, F) SVs resolved by long‐read sequencing. Green, *tremula*; purple, *alba*; blue, M147‐TRAs; yellow, *de novo* telomeres; lightning, breakpoint; arrow, T‐DNA insertion. Scale bar, 5 Mb.

Short‐read resequencing revealed large SVs in six of the 16 regenerated lines at 10 loci. These included deletions, arm‐loss and a segmental duplication spanning 130 Kb to 6.7 Mb (Figure 1B,C, Table S3). Four lines carried two SVs on homologous (*tremula* [T] and *alba* [A]) or non‐homologous chromosomes. Most SVs overlapped M147‐TRAs (blue ticks, Figure 1B), with a slight preference on chromosomes with higher target densities. An exception was line #7–3, which showed a 6.7 Mb arm‐loss on A14—a chromosome devoid of M147 (Zhou et al. 2023), likely reflecting collateral instability from extensive genome cleavage.

To expand structural diversity without sexual reproduction, five micropropagated (*m*) lines (#7–3, #7–4, #8–2, #8–4 and #8–6) were subjected to callus re‐induction and regeneration (Figure 1A). We recovered 22 re‐regenerated (*r*) plants (2–7 per line, Table S2). Plant growth was similar among wild type (WT) and transgenic lines regardless of culture history (Figure 1D, Figure S1). Resequencing showed stable parental profiles in micropropagated lines, whereas new SVs were detected in four re‐regenerated plants at a minimum of six loci (Table S3).

The new SVs included M147‐TRA deletions and duplications, arm‐loss, arm‐duplication and a whole‐chromosome duplication, ranging from 590 Kb to 9.9 Mb (Figure 1B,C, Table S3). Most events again overlapped or flanked M147‐TRAs, except the whole‐chromosome duplication of T14 in #7–3r1 in which homologous A14 was truncated. This event may represent a compensatory response to restore gene dosage in the parental line, though experimental validation is required.

To more thoroughly assess genome restructuring outcomes, we performed long‐read nanopore sequencing of two line‐pairs (#7–3 and #8–4), along with WT. Each pair comprised a parental and a re‐regenerated line, both derived from micropropagated material (Figure 1A), yielding five haplotype‐resolved genome assemblies (Table S4). Parental SVs identified using short‐read resequencing were confirmed, including two arm‐loss events on A14 and T11, both associated with *de novo* telomere formation. Estimated telomere lengths reached ~28 Kb on truncated A14 (SV1_#7–3_) and ~17 Kb on truncated T11 (SV1_#8–4_, Figure 1E,F, Table S5).

Three SVs uniquely associated with callus re‐induction were also confirmed, including two M147 duplications (A11 in #8–4r4 and A17 in #7–3r1) and a T14 whole‐chromosome duplication (#7–3r1, Figure 1C). The structural configuration of the duplicated T14—whether present as a free chromosome or fused to another—remained unresolved.

Long‐read assemblies further uncovered cryptic rearrangements missed by short‐read mapping. Both #7–3 and #7–3r1 harboured an inter‐chromosomal translocation between T08 and A03, with breakpoints mapped within M147‐TRAs, consistent with Cas9–mediated cleavage. This translocation produced two chimeric chromosomes of ~17 Mb (SV2_#7–3_) and ~25 Mb (SV3_#7–3_, Figure 1E).

Both #8–4 and #8–4r4 carried two translocations. The first involved homologous T10 and A10 with non‐syntenic breakpoints: the T10 breakpoint occurred between two M147‐TRAs, while the A10 breakpoint mapped to the distal long‐arm (Figure 1F). This translocation generated a 12.6 Mb chromosome (SV2_#8–4_), the smallest in the genome and a 32 Mb chromosome (SV3_#8–4_), the third largest. The second translocation, between T05 and A17, involved pericentromeric breakpoints (Figure 1F) and produced a ~25.7 Mb chromosome carrying two M147‐TRAs (SV4_#8–4_) and a 16.5 Mb M147‐free chromosome (SV5_#8–4_). The 16.5 Mb derivative contained a head‐to‐head insertion of two T‐DNA copies at the fusion (Figure 1F), suggesting that at least one breakpoint coincided with T‐DNA integration.

To further explore cryptic SVs, we performed low‐coverage long‐read sequencing of six #9‐lines (Table S2). In addition to confirming short‐read SV calls, we identified previously undetected SVs in three events. These included at least five translocations in #9–3, three in #9–7 and one in #9–36, which was indistinguishable from WT by short‐read sequencing (Tables S3 and S6). Overall, this platform generated diverse structural outcomes, with at least 43.8% (7/16) of lines harbouring SVs and up to seven distinct SVs in a single individual (Figure 1C). Given that only a limited number of events were examined by long‐read sequencing, the extent of SVs is likely underestimated.

Together, these results demonstrate that CRISPR targeting of satellite DNA can drive chromosome‐scale restructuring without sexual reproduction. Our data suggest these SVs remained stable during micropropagation, whereas dedifferentiation into mitotically active callus facilitated further Cas9–mediated genome restructuring. While currently stochastic, this study establishes a proof‐of‐concept for chromosome engineering at scale. This work also provides a germplasm resource for studying chromosome dosage and genome plasticity in long‐lived, clonally propagated species. The widespread occurrence of satellite DNA in eukaryotic genomes suggests that this strategy may be broadly applicable across species.

## Author Contributions

C.‐J.T. and R.Z. designed research; M.S.S.C., M.R.D., K.M. and K.Y. performed experiments; R.Z. analysed data, assisted by B.V. and C.R.B. R.Z. and C.‐J.T. wrote the manuscript with input from all authors.

## Funding

This work was supported by the Georgia Research Alliance, Georgia Seed Development and University of Georgia.

## Conflicts of Interest

The University of Georgia Research Foundation has filed a patent application on behalf of R.Z. and C.‐J.T. All other authors declare no competing interests.

## Supporting information


**Tables S1–S7** and

**Figure S1**.

## Data Availability

Sequencing data are available at NCBI BioProject PRJNA1469888.
