# Supplementary material for: Satellite DNA Editing Enables Multiplexed Chromosome Restructuring in Populus
Source: Plant Biotechnol J. 2026 Aug 24;24(11):5830–2. doi: 10.1111/pbi.70741 (PMC13501387; doi:10.1111/pbi.70741)
Supplement: Supplementary file 1 [file PBI-24-5830-s001.pdf]

Supporting Information

Satellite DNA Editing Enables Multiplexed Chromosome Restructuring in *Populus*

Ran Zhou[1,2,3,4], Margot S.S. Chen[1], MaKenzie R. Drowns[1,4], Kathrine Mailloux[5], Kangquan Yin[1,4], Brieanne Vaillancourt[5], C. Robin Buell[4,5,6,7], Chung-Jui Tsai[1,2,3,4,*]

[1]Warnell School of Forestry and Natural Resources, University of Georgia, Athens, GA, USA
[2]Department of Genetics, University of Georgia, Athens, GA, USA
[3]Department of Plant Biology, University of Georgia, Athens, GA, USA
[4]The Plant Center, University of Georgia, Athens, GA, USA
[5]Center for Applied Genetic Technologies, University of Georgia, Athens, GA, USA
[6]Institute of Plant Breeding, Genetics, and Genomics, Athens, GA, USA
[7]Department of Crop and Soil Sciences, University of Georgia, Athens, GA, USA
* Corresponding author: cjtsai@uga.edu

Content

Methods
Figure S1. Representative tissue-culture growth of transgenic and control lines
Table S1. M147-TRA size and predicted gRNA target site numbers
Table S2. Transgenic lines and associated analyses
Table S3. Structural variants detected by short-read whole-genome resequencing
Table S4. Nanopore long-read sequencing and genome assembly metrics
Table S5. Telomeric repeat tracts on chromosomes T11 and A14 in wild-type and transgenic lines
Table S6. Split-read evidence from low-coverage Nanopore sequencing supporting translocation breakpoints
Table S7. Primers used in this study

Materials and Methods

Plant transformation and maintenance

Two guide RNAs (gRNAs) were designed to target the consensus sequence of the M147 satellite repeat family: M147.g7 (GGCAACCGGGGCATCGAAGTGGG, protospacer adjacent motif underlined) and M147.g8 (GTCAAAGCGTTTCGCCGTTTGCGG). A pair of oligos containing vector homology overhangs (Table S6) was assembled into pORE303N (Ortega et al., 2023). *Agrobacterium tumefaciens*-mediated transformation of *Populus tremula* × *P. alba* (INRA 717-1B4) was conducted as described (Bewg et al., 2022; Ortega et al., 2023) using 15 leaf discs in the first round or 45 in a repeated trial with the M147.g8 construct. Callus growth appeared normal, but callus recovery was markedly reduced for these constructs. Shoot regeneration, elongation, and rooting responses were much slower, with recovery of five rooted plants (two from M147.g7 and three from M147.g8) occurring 9–10 months after transformation compared to 4–5 months for unrelated constructs initiated at the same time. Once rooted, plant growth and subsequent vegetative propagation were indistinguishable from wild-type (WT) and other transgenic lines. Young leaves were collected and stored at −80 °C for DNA extraction and whole-genome resequencing.

Plants were maintained by vegetative propagation either through direct rooting of the shoot top on ½ MS or on root induction media (RIM, ½ MS supplemented with 0.01 mg/L indole-3-butyric acid [IBA], 0.2 g/L L-glutamine, and 0.25 g/L 2-(N-morpholino)ethanesulfonic acid [MES]), or via induction of axillary shoots from the main stem on shoot elongation media (SEM, MS supplemented with 0.05 mg/L benzylaminopurine [BA], 0.2 g/L L-glutamine, and 0.25 g/L 2-(N-morpholino)ethanesulfonic acid [MES]) (Ortega et al., 2023).

Plants for callus re-induction experiments (#7-3, #7-4, #8-2, #8-4, and #8-6) were micropropagated (*m*) for ~8 months. Leaf discs were cultured on callus induction media (CIM, MS supplemented with 10 μM 1-naphthaleneacetic acid [NAA], 5 μM N^6-2-isopentenyladenine [2ip], 0.2 g/L L-glutamine, and 0.25 g/L MES) and subcultured biweekly for 4–8 weeks. Micro-calli emerging from the leaf discs were physically separated and tracked as distinct re-regeneration (*r*) events for shoot induction (MS supplemented with 0.05 mg/L thidiazuron [TDZ], 0.2 g/L L-glutamine, and 0.25 g/L MES), followed by shoot elongation and root

induction. Rooted plants were obtained in 3–4 months for 2–7 re-regenerated lines per parental line, totally 22 lines. Young leaves from all 22 regenerants and their micropropagated parental lines (5 total) were collected for genomic DNA extraction.

For nanopore long-read sequencing, stem segments from micropropagated parental lines #7-3 and #8-4 (over 20 months) and re-regenerated #7-3r1 and #8-4r4 lines (8–9 months) were cultured on SEM to promote axillary shoot growth. Approximately 50 newly emerged leaves were collected and stored at −80 °C for high molecular weight (HMW) genomic DNA extraction. Similar sampling was conducted for micropropagated parental lines #9-3, #9-5, #9-7, #9-11, #9-12, #9-36, and WT for low-coverage long-read sequencing.

DNA extraction, Illumina whole-genome sequencing, and data analysis

Genomic DNA for Illumina whole-genome resequencing was extracted from young leaves using the plant DNA minipreparation protocol (Dellaporta et al., 1983). DNA libraries were prepared using the sparQ DNA Fragment & Library Kit (QuantaBio) with NEBNext Multiplex Dual Index UMI Adaptors (NEB) and sequenced on an Illumina NovaSeq (paired-end 150 bp) at BMKGENE or via the Georgia Genomics and Bioinformatics Core, University of Georgia (RRID:SCR_010994). We obtained 6–7 million reads per sample for the first group of transgenic plants (#7 and #8 lines), 14–28 million reads per sample for the repeated transformation (#9 lines), and 3–28 million reads per sample from the callus re-induction experiment.

Raw reads were trimmed for adapter sequences and quality-filtered using trim_galore in Cutadapt (Martin, 2011). The cleaned reads were aligned to the *Populus tremula* × *P. alba* INRA 717-1B4 (717) reference genome (Zhou et al., 2023) using BWA-MEM (Li, 2013). Duplicate reads were marked using MarkDuplicates (Picard) in GATK (Van der Auwera et al., 2013). To quantify genome-wide read depth, the bamCoverage module from the deepTools2 suite (v3.5.1) (Ramírez et al., 2016) was used with a 10-Kb bin size across all 38 chromosomes of the two haplotypes. Normalization was disabled (--normalizeUsing None) to capture absolute coverage variation and facilitate detection of large-scale chromosomal deletions. A persistent >50% reduction in coverage relative to the WT control was classified as a deletion. Putative breakpoint

junctions were manually inspected in IGV (Integrative Genomics Viewer) (Thorvaldsdóttir et al., 2013).

HMW DNA extraction, nanopore sequencing, genome assembly, and data analysis

HMW genomic DNA was extracted from apical leaves using the NucleoBond HMW DNA isolation kit (Takara). DNA purity and integrity were assessed by NanoDrop spectrophotometry and gel electrophoresis. Sequencing libraries were prepared using the Ligation Sequencing Kit V14 (Oxford Nanopore Technologies) according to the manufacturer's instructions and sequenced on FLO-MIN114 flow cells (one sample per flow cell) using a MinION device. For low-coverage sequencing, genomic libraries were prepared using the Native Barcoding Kit 24 V14 and sequenced on MinION flow cells (2-3 samples per flow cell). Basecalling was performed using Dorado (v1.3.1, https://github.com/nanoporetech/dorado) with the super-accuracy (SUP) model for R10.4.1 (Kit14) data. Reads with Q-score <10 were filtered and adapter and primer sequences were trimmed. Demultiplexing was performed using Dorado demux on the base called bam file with the --no-classify option.

De novo genome assembly was conducted using hifiasm with the -ont flag to generate high-continuity contigs. RagTag2 v2.1.0 (Alonge et al., 2022) was used to organize contigs into chromosomal-scale scaffolds. The ragtag.py *correct* module was first applied to identify and resolve potential misassemblies using the 717 reference genome (Zhou et al., 2023) as a guide. The bam file generated from RagTag was used for visual inspection of breakpoints or other regions of interest, such as T-DNA insertion sites. Pairwise alignment (paf) files generated by minimap2 (Li, 2018) were used to visualize assemblies relative to the 717 reference.

Tandem Repeats Finder (Benson, 1999) v4.09.1 was used to identify telomeric regions and *de novo* capping events for all five nanopore assemblies. Analyses were performed using a match weight of 2, mismatch penalty of 5, and indel penalty of 7, with match and indel probabilities set to 80 and 10, respectively, a minimum alignment score of 50 and a maximum period size of 2,000 bp. The output was screened for the canonical plant telomeric motif $(TTTAGGG)_n$ and its reverse complement. These repeat arrays were used to define chromosomal termini.

Structural variant identification from low-coverage data

Structural variants (SVs) were identified from low-coverage long-read sequencing data using Sniffles2 with default parameters (Sedlazeck et al., 2018). Homozygous breakend (BND) variants representing potential translocations or complex rearrangements were extracted (zgrep "BND" | grep "1/1") for downstream validation. BND variants present in the WT control sample were excluded to filter background variants and alignment artifacts. Variants located within regions of abnormally high read depth, likely resulting from unresolved repetitive sequences in the reference assembly, were also removed. Candidate BND variants were then manually inspected and validated using Integrative Genomics Viewer (IGV, v2.12.3) (Robinson et al., 2023). Supporting reads were extracted by read ID using seqtk subseq (https://github.com/lh3/seqtk) and aligned to the 717 reference genome, with the pORE303N vector sequence included, using minimap2 (Li, 2018). Only high-confidence split-read alignments (MAPQ = 60) spanning breakpoint junctions were retained as supporting evidence (Table S6). The supporting long-read sequences have been deposited in figshare (Zhou et al., 2026).

References

Alonge, M., Lebeigle, L., Kirsche, M., Jenike, K., Ou, S., Aganezov, S., Wang, X., Lippman, Z.B., Schatz, M.C. and Soyk, S. (2022) Automated assembly scaffolding using RagTag elevates a new tomato system for high-throughput genome editing. *Genome Biol* **23**, 258.

Benson, G. (1999) Tandem repeats finder: a program to analyze DNA sequences. *Nucleic Acids Research* **27**, 573-580.

Bewg, W.P., Harding, S.A., Engle, N.L., Vaidya, B.N., Zhou, R., Reeves, J., Horn, T.W., Joshee, N., Jenkins, J.W., Shu, S., Barry, K.W., Yoshinaga, Y., Grimwood, J., Schmitz, R.J., Schmutz, J., Tschaplinski, T.J. and Tsai, C.-J. (2022) Multiplex knockout of trichome-regulating MYB duplicates in hybrid poplar using a single gRNA. *Plant Physiology* **189**, 516-526.

Dellaporta, S.L., Wood, J. and Hicks, J.B. (1983) A plant DNA minipreparation: Version II. *Plant Mol Biol Rep* **1**, 19-21.

Li, H. (2013) Aligning sequence reads, clone sequences and assembly contigs with BWA-MEM. *arXiv: Genomics*.

Li, H. (2018) Minimap2: pairwise alignment for nucleotide sequences. *Bioinformatics* **34**, 3094-3100.

Martin, M. (2011) Cutadapt removes adapter sequences from high-throughput sequencing reads. *EMBnet.journal* **17**, 3.

Ortega, M.A., Zhou, R., Chen, M.S.S., Bewg, W.P., Simon, B. and Tsai, C.-J. (2023) In vitro floral development in poplar: insights into seed trichome regulation and trimonoecy. *New Phytologist* **237**, 1078-1081.

Ramírez, F., Ryan, D.P., Grüning, B., Bhardwaj, V., Kilpert, F., Richter, A.S., Heyne, S., Dündar, F. and Manke, T. (2016) deepTools2: a next generation web server for deep-sequencing data analysis. *Nucleic Acids Research* **44**, W160-W165.

Robinson, J.T., Thorvaldsdottir, H., Turner, D. and Mesirov, J.P. (2023) igv.js: an embeddable JavaScript implementation of the Integrative Genomics Viewer (IGV). *Bioinformatics* **39**, btac830.

Sedlazeck, F.J., Rescheneder, P., Smolka, M., Fang, H., Nattestad, M., von Haeseler, A. and Schatz, M.C. (2018) Accurate detection of complex structural variations using single-molecule sequencing. *Nat Methods* **15**, 461-468.

Thorvaldsdóttir, H., Robinson, J.T. and Mesirov, J.P. (2013) Integrative Genomics Viewer (IGV): high-performance genomics data visualization and exploration. *Briefings in Bioinformatics* **14**, 178-192.

Van der Auwera, G.A., Carneiro, M.O., Hartl, C., Poplin, R., del Angel, G., Levy-Moonshine, A., Jordan, T., Shakir, K., Roazen, D., Thibault, J., Banks, E., Garimella, K.V., Altshuler, D., Gabriel, S. and DePristo, M.A. (2013) From FastQ Data to High-Confidence Variant Calls: The Genome Analysis Toolkit Best Practices Pipeline. *Curr Protoc Bioinformatics* **43**, 11.10.11-11.10.33.

Zhou, R., Chen, M., Mailloux, K., Vaillancourt, B., Buell, C.R. and Tsai, C.-J. (2026) Nanopore reads supporting split alignments at putative translocation breakpoints in satellite DNA-edited *Populus tremula* × *P. alba*. In: *figshare.* *https://doi.org/10.6084/m9.figshare.33019193*.

Zhou, R., Jenkins, J.W., Zeng, Y., Shu, S., Jang, H., Harding, S.A., Williams, M., Plott, C., Barry, K.W., Koriabine, M., Amirebrahimi, M., Talag, J., Rajasekar, S., Grimwood, J., Schmitz, R.J., Dawe, R.K., Schmutz, J. and Tsai, C.-J. (2023) Haplotype-resolved genome assembly of *Populus tremula* × *P. alba* reveals aspen-specific megabase satellite DNA. *Plant Journal* **16**, 1003-1017.

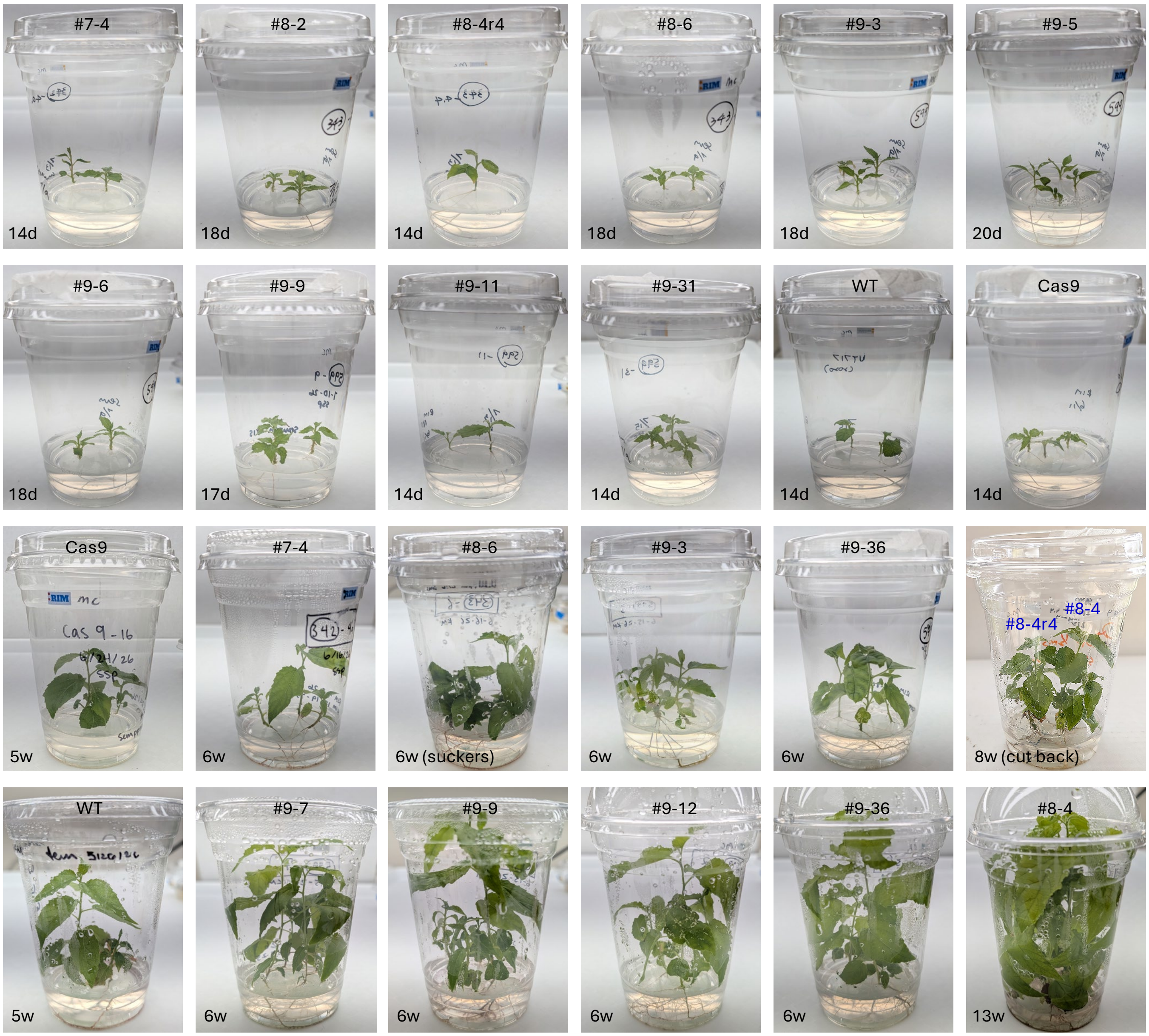

Figure S1. Representative tissue-culture growth of transgenic and control lines.
Representative images show multiple independent transgenic events and controls at different stages of tissue culture, including newly rooted and well-established plants. Numbers indicate time in culture (days or weeks) at imaging.
